# Sport and Exercise Psychology Studies in Brazil: Performance or Health?

**DOI:** 10.3389/fpsyg.2019.02154

**Published:** 2019-09-25

**Authors:** Lenamar Fiorese, André Luiz Felix Rodacki, Nayara Malheiros Caruzzo, Caio Rosas Moreira, Andressa Ribeiro Contreira, Aline Mendes de Lima, Leonardo de Sousa Fortes, João Ricardo Nickenig Vissoci, Joice Mara Facco Stefanello

**Affiliations:** ^1^Department of Physical Education, State University of Maringá, Maringá, Brazil; ^2^Biological Sciences Sector, Federal University of Paraná, Curitiba, Brazil; ^3^Duke Global Health Institute, Duke University, Durham, NC, United States; ^4^Department of Physical Education, Federal University of Paraíba, João Pessoa, Brazil; ^5^Emergency Medicine, Department of Surgery, Duke University, Durham, NC, United States; ^6^Duke Global Neurosurgery and Neurology, Department of Neurosurgery, Duke University, Durham, NC, United States; ^7^Department of Physical Education, Federal University of Paraná, Curitiba, Brazil

**Keywords:** graduate education, exercise and sport sciences, psychology, sport, exercise

## Abstract

The low professional insertion of psychologists in Brazilian sports teams, as well as the Sport Psychology course being seldom offered in undergraduate Psychology courses, may reflect in the current scenario of scientific research in Brazil. It is still not clear what Graduate Programs (GPs) have adopted directions regarding the development of studies in Sport and Exercise Psychology (SEP) research fields since an assessment or mapping of scientific knowledge production in this area has not yet been performed involving Exercise and Sport Science and Psychology GPs. This study aimed at investigating institutional research and their themes in SEP from these graduate programs. Studies were selected and retrieved from a national database (Sucupira Platform), that contains all registered researches from all Exercise and Sport Science (*n* = 31) and Psychology (*n* = 84) GPs in the country. Data were analyzed through R software using text mining techniques, latent semantic analysis and K-means clustering. Results revealed that research involving SEP is predominantly being developed at Exercise and Sport Science GPs (*n* = 171; *p* < 0.01) in comparison to psychology GPs (*n* = 39), mostly located in the south and southeast regions of Brazil. This research has focused on the effects of physical exercise and quality of life, while Psychology GPs have analyzed sport as associated with health and education, as a way to promote social support and to study sports’ psychological aspects. It was concluded that Exercise and Sport Science GPs had the most significant contribution to SEP. Investigations were focused on the interface of exercise with quality of life, health and education, with gaps existing in programs advancing in the studies on sports and performance.

## Introduction

Sport and Exercise Psychology (SEP) in Brazil dates back to about 50 years ago. Since then, this area has been seeking to organize its professional field (Queiroz et al., 2016) and experienced an expressive growth from 2010 due to the presence of SEP professionals in elite sports, as well as the increase in scientific research in Brazil (Vieira et al., 2010, 2013). However, a more pronounced SEP practical development has been hindered by the lack of specific knowledge and difficulties to make research results available for SEP professionals (Aoyagi et al., 2012). There is a large number of sport psychology professionals that are still unaware of the benefits of scientific research as a tool to improve performance (Ucha, 2008). Besides, there is a lack of studies devoted to analyzing mental, emotional and behavioral aspects involved in sports performance (Stefanello, 2009). This is contrasting with the achievements of some athletes and teams in major international competitions.

The Brazilian SEP publications are closely related to the development of graduate programs (GPs) (Vieira et al., 2013), where the more experienced researchers are based, and the more robust studies are carried out. It is interesting to observe that most SEP studies are conducted in Exercise and Sport Science GPs rather than Psychology ones. This may be related to the lack of SEP as a formal discipline in undergraduate Psychology courses and may explain the modest scientific contribution and the reduced number of Sport Psychologists involved in high-performance sports (Vieira et al., 2010). This opposes the directions recommended by the APA’s Division 47 (Exercise and Sport Psychology), which advocates in favor of research and practice unification (Aoyagi et al., 2012), as a strategy to increase the professionalization of sport psychologists.

Mapping the intellectual products related to SEP may constitute an attractive strategy to identify the way the researchers are organized and how specific academic themes advance and are structured. This may be relevant in many developing countries where SEP is a new professional field (Queiroz et al., 2016). For instance, it has been identified that Sport and Exercise Science GPs publish more SEP studies than their Psychology GP counterparts (Vieira et al., 2013). In addition, it is not clear the research emphasis or trends applied by Exercise and Sport Science and Psychology GPs regarding different SEP practical and theoretical approaches (e.g., physical activity and health; physical exercise, elderly and chronic diseases; sport, stress and motivation; sport and exercise for child and adolescent development, etc.). Thus, understanding how the scientific research is produced, organized and structured is relevant not only to the development and trends for high performance sports in Brazil but also to allow future comparisons between countries with similar academic organizations and sport’s approaches.

Therefore, this study aimed to analyze the approaches applied by Exercise and Sport Science and Psychology GPs as they respond for most thesis and dissertations SEP produced in the country. The geographic distribution of these GPs was also provided.

## Materials and Methods

**FIGURE 1:**
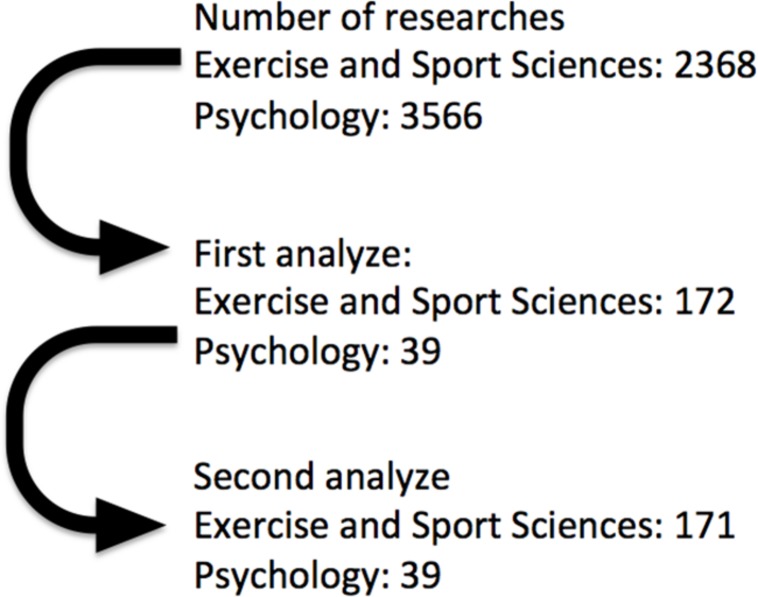
Research selection flowchart.

All studies performed at Brazilian GPs in Exercise and Sport Sciences (*n* = 31) and Psychology (*n* = 84) were included in the analysis. The studies containing words related to SEP in the title were selected for further analysis. Research titles were retrieved from the Sucupira Platform^1^, which is a governmental, but public domain tool developed for the National assessment of the GPs from 2013 to 2016. Three researchers performed the analysis of all studies conducted by the Exercise and Sport Sciences (*n* = 2368 studies) and Psychology (*n* = 3566 studies) GPs. Disagreements were resolved by consensus (Figure 1).

All data used was obtained from publicly available data sources from documents used in research. No human subjects were enrolled and no humans subject data were used. The present study used only documental data available in Sucupira Platform, therefore, it had no human subjects, requiring no participants’ consent, nor ethical approval as define by the Brazilian national research regulation CNS 196/96.

### Data Analysis

The SPSS software (version 24.0) was used to verify the difference in researches’ distribution across Exercise and Sport Science and Psychology GPs through the Chi-Square test. To identify the main themes appearing in the GP’s research titles a combination of text mining, latent semantic analysis (LSA) and clustering techniques was used. Text mining is consisted of extracting content from text based on meaning and context; in other words, it gathers structured information from a non-structured text (Feinerer et al., 2018). This analysis comprises three steps: (a) preprocessing of the documents; (b) extracting and grouping text patterns; and (c) content evaluation (Rezende et al., 2011). The document processing step was conducted by transforming all letters to lowercase; stemming; removing stopwords, accents, punctuation, numbers and exceeding spaces. Then, each word was extracted and vectorized. The terms originated from text mining were quantified for their title frequency. With the vectorized data, a LSA was applied to identify similarities in the semantic content between titles. Similarities were generated based on a weighted score attributed to each document word, which was derived from their appearance across documents. Based on these weights, titles were scored, and similarities were defined by title association (Landauer, 1998). After calculating the similarities between titles, a K-means cluster analysis approach was used to identify the main themes arising from the titles. The number of clusters was defined by screen plot visualization, model accuracy (AIC, BIC and EBIC statistics), class membership (classes deemed acceptable if having at least 5% of cases) and theoretical coherence.

Within each cluster, the term association was calculated from a polychoric correlation matrix and depicted as an undirected network graph. The use of an algorithm defined the networks to plot nodes (terms) according to their association. Thus, nodes with higher association were positioned closer to each other. Edges connect nodes, and the thickness (color intensity) of theses edges reflects the strength of the association between nodes. The nodes represent variables and the edges represent the connection between variables (i.e., words). Thus, central nodes have strong connections with other nodes, while peripheral nodes indicate fewer connections. The thickness of the edges reflects the associations’ strength.

The main analyses were conducted using the R software (version 3.2). Text mining was conducted using the “TM” package (version 0.5.1). Latent semantic analysis was performed using the “LSA” package (version 0.73.1). Clusters were modeled using the “skmeans” (version 0.2-11). Visualizations were built using “qgraph” (version 1.6.1), wordcloud (version 2.6) and ggplot2 (version 3.0.0) packages.

## Results

### The Scenario of the SEP Related Research

**FIGURE 2:**
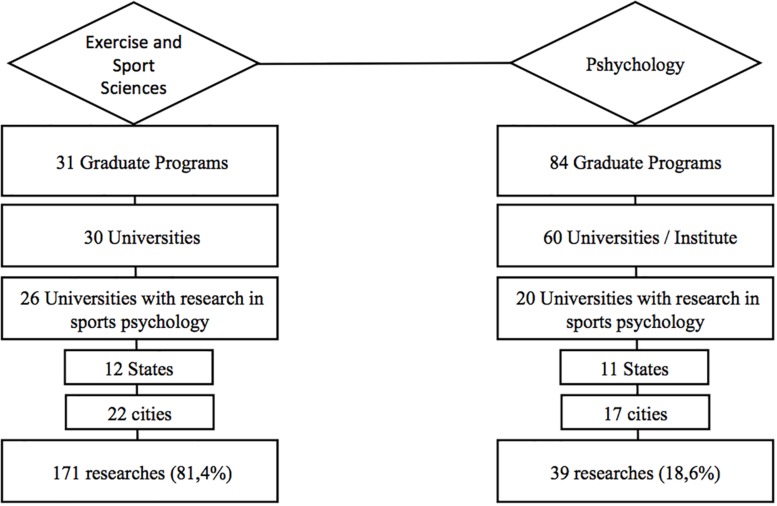
Distribution of institutional researches in Sport and Exercise Psychology from Exercise and Sport Science and Psychology graduate programs in Brazil.

Figure 2 shows the distribution of SEP researches in all Brazilian Exercise and Sport Science and Psychology GPs. The existence of 84 psychology programs represents more than twice the number of Exercise and Sport Science programs (*n* = 31 programs). Despite such discrepancy, a vast majority of the studies (*n* = 171; 81.4%; *p* < 0.01) were conducted in the Exercise and Sport Science GPs, in comparison to the Psychology GPs (*n* = 39; 18.6%).

### SEP Related Researches Main Themes

**FIGURE 3:**
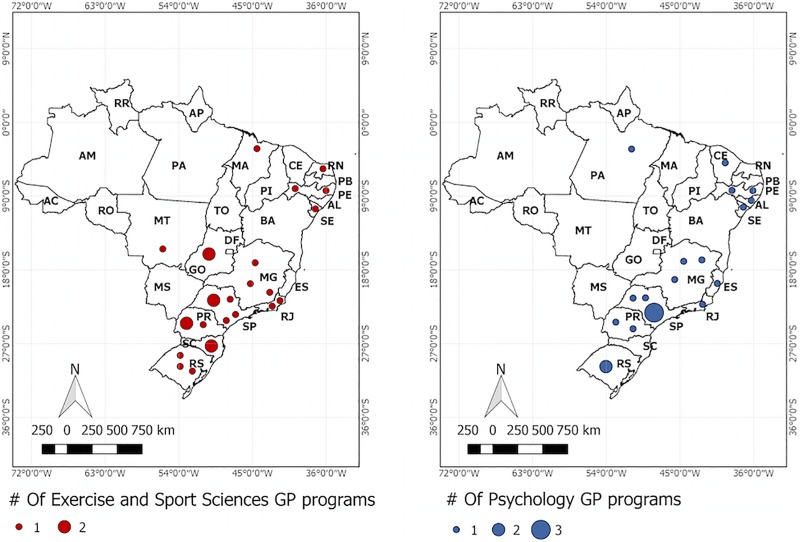
Distribution of Exercise and Sport Science and Psychology GPs with Sport and Exercise Psychology researches in Brazilian cities.

The geographic distribution of Exercise and Sport Science (*n* = 26 universities) and Psychology programs (*n* = 20 universities) with SEP researches are shown in Figure 3 and indicated predominance in the south and southeast regions. The number of programs in the North region is scarce and only one Psychology GP was identified, while no GPs were identified in the country’s central area. Two Exercise and Sport Science programs were based on the central region.

**FIGURE 4:**
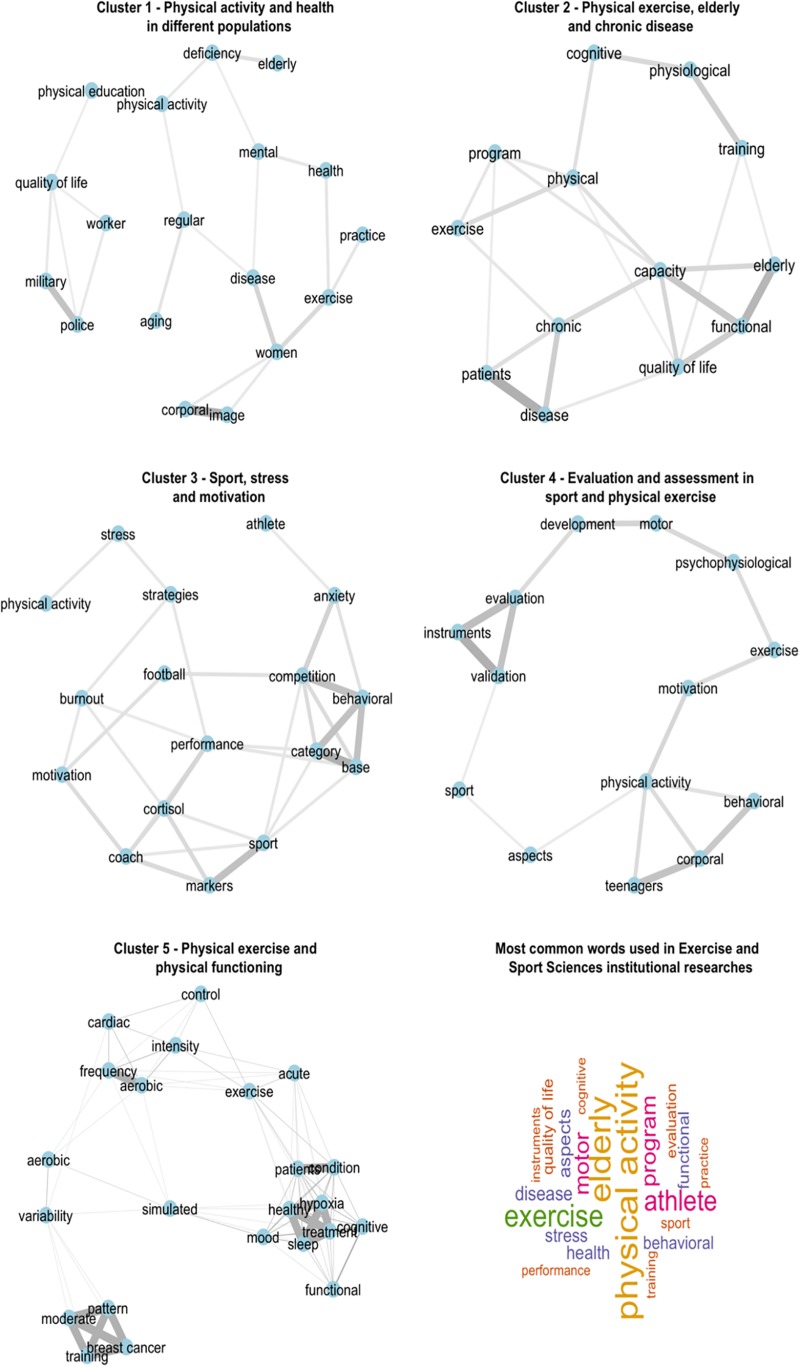
Correlation network and a word cloud of title words of Exercise and Sport Science GPs.

**FIGURE 5:**
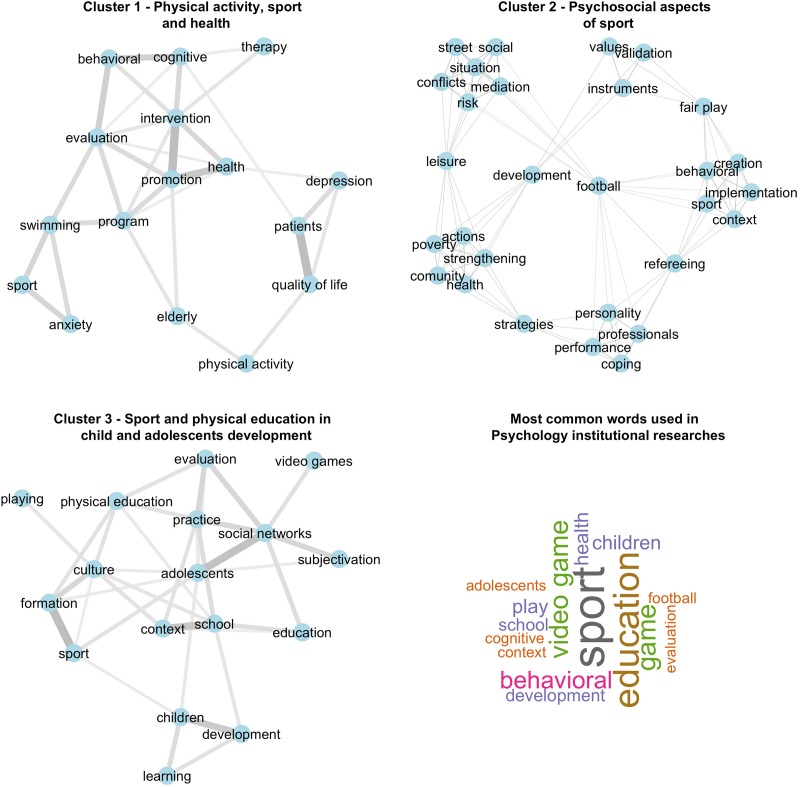
Correlation network and a word cloud of title words of Psychology GPs.

The cluster analysis resulted in five clusters for Exercise and Sport Science GPs (Figure 4) and three clusters for Psychology GPs (Figure 5). Following the cluster identification, a network analysis was conducted using the word groups from each cluster to reveal the main SEP research themes. The network analysis allowed the identification of each cluster’s profile and a network graphing for each cluster. The categorization for the Exercise and Sport Science resulted in the following clusters: Cluster 1 = Physical activity and health in different populations; Cluster 2 = Physical exercise, elderly and chronic disease; Cluster 3 = Sport, stress and motivation; Cluster 4 = Evaluation and assessment in sport and physical exercise; Cluster 5 = Physical exercise and physical functioning. Researches from Psychology GPs were categorized as follows: Cluster 1 = Physical activity, sport and health; Cluster 2 = Psychosocial aspects of sport; Cluster 3 = Sport and physical education in child and adolescent development.

#### Exercise and Sport Sciences

Resulting network from cluster 1, Physical activity and health in different populations, present institutional researches involving themes associating physical activity practice to health and quality of life improvements for a variety of populations. The terms “exercise,” “practice” and “health” were positioned in the center of the network, and peripheral nodes were related to the different investigated populations, such as “elderly,” “women,” “workers,” “military/policemen” and “individuals with disabilities.”

Cluster 2 network, Physical exercise, elderly and chronic disease, presented the term “physical” as a central node, being connected to terms of considerable benefits such as “cognitive,” “physiological” and “functional capacities.” It was also possible to identify significant associations of exercise and two groups, one involving “quality of life” and “functional capacity in elderly” research, and the other involving “physical exercise” related to the treatment of “patients with chronic disease.”

Cluster 3 network, Sport, stress and motivation, represented psychological factors in sports. In this network, the theme “sport” is the central axis, connected to two main peripheral terms “base category” and “markers of cortisol.” Research involving “base category” studied “anxiety at competition” and “behavioral aspects” of young athletes. For “performance,” it was found connections with psychophysiological factors (“cortisol”), and a variety of psychological variables such as “coping strategies,” “burnout,” “stress” and “motivation.” Furthermore, the term “sport” was positioned closer to the term “coaches” than “athletes,” with “football” (soccer) being only highlighted sport among these studies.

Cluster 4 network, Evaluation and assessment in sport and physical exercise, comprised researches related to the “evaluation,” “validation” and “development” of psychometric instruments to be used for analysis with sport and exercise. These aspects may indicate that Physical Education GPs dedicate a part of their institutional researches to the transcultural adaptation of instruments and the development of assessment tools to be used in SEP research.

Cluster 5 network, Physical exercise and physical functioning, show different factors related to health/sickness and their associations with physical activity. Significant relationships were observed in the top node that referred “exercise” as a tool for treating diseases such as a “cognitive treatment,” showing positive relationships with treating of “hypoxia,” better quality of “sleep,” which represent the physical exercise’s positive impact over mental health aspects in the treatment of “stress” and “mood state.” The third group of nodes showed a “moderate training” as a tool for “breast cancer” treatment.

#### Psychology

Cluster 1 network for programs in Psychology presents sport and exercise and its association with health. As observed, the term “health” was highlighted in the network and is related to the assessment of “behavioral” aspects of health as well as the “promotion” of health. The terms “elderly,” “physical activity” and “quality of life” were connected, showing that quality of life improvement for the elderly population seems to be the focus of some of these investigations involving physical activity.

Cluster 2 network, Psychosocial aspects of the sport, it was observed that “football” has been the main focus of studies of specific sports. The association of “sport” as a “leisure” activity was seen in the close positioning of “social aspects,” “conflict mediation,” and “street situation” terms, these nodes were connected to “areas of risk” and social vulnerability contexts, showing the interest for these phenomena in Psychology GPs. The interest in the “development” of “validation” and “instruments” to assess psychological aspects of emotional control and performance in athletes was also observed. On the other hand, “development” has shown a connection with “strengthening,” “community,” “health” and “poverty” which are possibly receiving “actions” and “strategies” through “leisure” sports. These themes indicate the importance of educating young athletes and preparing them for career transition in sport.

Cluster 3 network, Sport and physical education in child and adolescent development, presents the “educational context” that has been peripherally positioned showing the sport as a “ludic” practice used in education and “learning” to develop “children” and “adolescents” in schools. Words such as “formation,” “culture” and “development” as centralized in the network reveals the interest in the interface between sports and physical education in the developmental process, primarily when related to innovative terms like “video games” or “social networks.”

**FIGURE 6:**
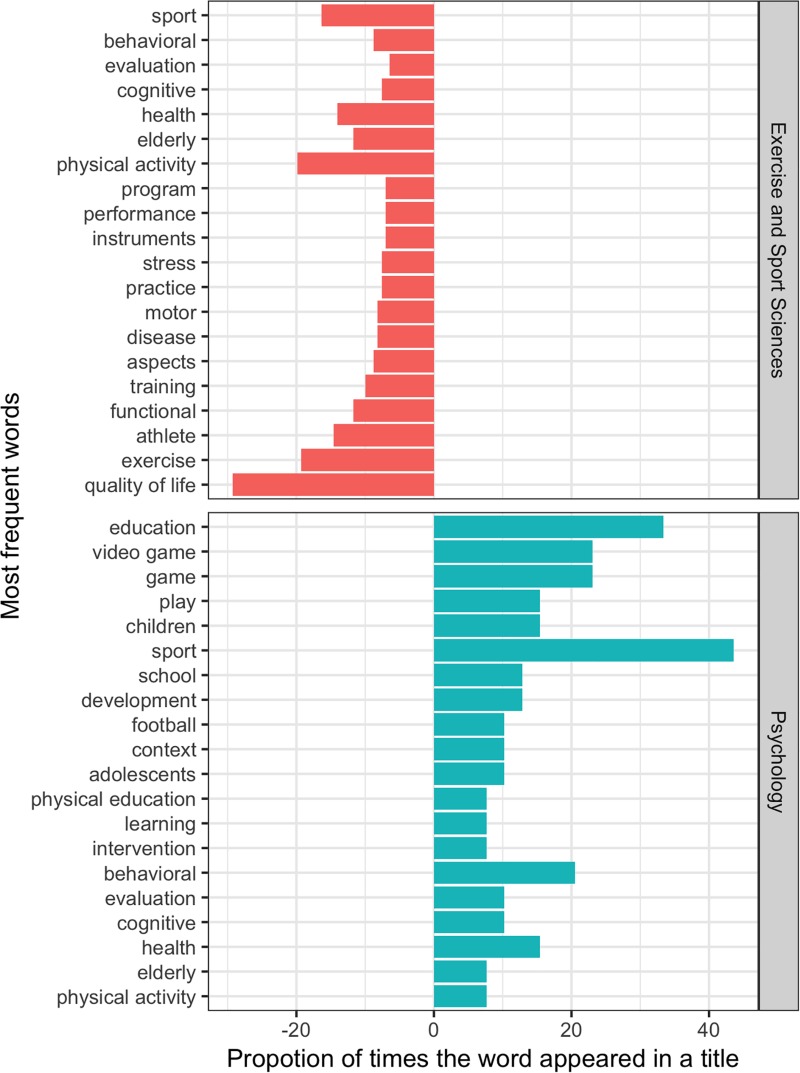
Frequent words and the title word frequency in Exercise and Sport Science and Psychology GPs.

The distribution of the frequent title words in Exercise and Sport Science and Psychology GPs are shown in Figure 6 and Table 1.

**TABLE 1:** Keyword frequency for the Exercise and Sport Science and Psychology GPs clusters.

**Exercise and sport science**	**Psychology**
**Cluster 1 – Physical activity and health in different populations (*n* = 105)** (26) Quality of Life (24) Physical Activity (19) Health (8) Elderly (5) Aging – Exercise – Military – Practice (4) Deficiency – Disease **Cluster 2 – Physical exercise, elderly and chronic disease (*n* = 135)** (20) Quality of Life (15) Exercise (11) Functional (9) Disease – Elderly – Training (7) Patients – Program (6) Capacity – Cognitive – Physical (5) Chronic – Physiological (4) Aspects – Behavioral – Mental – Stress – Women **Cluster 3 – Sport, stress and motivation (*n* = 35)** (13) Athletes (9) Performance (8) Football (5) Anxiety **Cluster 4 – Evaluation and assessment in sport and physical exercise (*n* = 168)** (24) Sport (11) Aspects (10) Instruments – Motor (8) Behavioral – Evaluation – Exercise (7) Corporal – Physical Activity – Teenagers (6) Development – Motivation – Psychophysiological – Validation (5) Context – Image – Performance – Practice (4) Children – Coach – Perception – Social – Stress – Training **Cluster 5 – Physical exercise and physical functioning (*n* = 24)** (6) Functional (5) Cardiac – Exercise (4) Cognitive – Humor	**Cluster 1 – Physical activity, sport and health (*n* = 51)** (6) Sport (5) Behavioral (4) Cognitive – Health (3) Elderly – Intervention – Quality of life (2) Anxiety – Depression – Evaluation –Patient – Physical activity – Program – Promotion – Swimming – Therapy (1) Adjuvant – Adults – Apprentices – Physical Aptitude **Cluster 2 – Psychosocial aspects of Sport (*n* = 28)** (6) Sport (3) Football (2) Behavioral – Development – Fair play – Leisure – Refereeing – Strategies (1) Actions – Application – Community – Conflicts – Context – Coping – Performance **Cluster 3 – Sport and physical education in child and adolescents development (*n* = 65)** (13) Education (9) Videogame (6) Playing (5) Children – Sport (4) Adolescents – School (3) Context – Learning – Physical Education (2) Culture – Cyber bulling – Development – Evaluation – Formation

## Discussion

This study aimed to analyze SEP research, from Exercise and Sport Science and Psychology graduate programs in Brazil, which provided a better identification of trends and the SEP research perspectives in emerging countries and other countries using similar approaches for further comparisons. The results showed that the majority of scientific interest in SEP is developed by Exercise and Sport Science programs rather than Psychology ones. As a consequence, the most frequently studied themes are related to the relationship between physical exercise and quality of life.

The higher prevalence of Exercise and Sport Science researches in the field of SEP (Figure 2) may reflect a low legitimacy attributed to psychologists. Indeed, SEP is one of the youngest branches of psychological sciences, which was officially recognized in 1965 (Brandão, 2007) and regulated as a professional area only in 2000 (Vieira et al., 2013). Although it is a new area of study, no substantial changes were introduced in the curricular structure of undergraduate psychology courses and in a few cases, SEP disciplines are offered as elective. On the other hand, SEP is regularly provided in Exercise and Sport Science undergraduate programs. It is plausible that the little effort to provide a strong background in sport and physical activity may account for these differences. Therefore, it is not difficult to understand why Exercise and Sport Science graduate programs present a larger number of SEP publications than Psychology counterparts (Vieira et al., 2013).

The geographical distribution of Exercise and Sport Science and Psychology graduate programs with SEP researches indicates a large concentration in the South and Southeast regions, with a few ones located in the remaining areas (Figure 3). These findings are aligned with the results presented by Vilarino et al. (2017) that reported 41.4 and 31.3% of the SEP research groups are based in the Southeast and South regions, respectively. This is further reinforced as most scientific papers related to SEP are published in national journals are from these regions (Andrade et al., 2015). Therefore, theses finds contributes to identifying where the SEP is not having been studied and to indicate where new efforts and investments should must do to grow the body of researchers in SEP.

The main themes in Exercise and Sport Science programs included exercise and quality of life (Figures 4, 6), followed by studies in sports. The Exercise and Sport Science programs responded by 81.4% of all studies involving SEP performed by the GPs (Figure 2). The main focus of these studies was related to the understanding of the association between exercise and physical activity, well-being and quality of life (Queiroz et al., 2016).

On the other hand, Psychology programs responded by 18.6% of all SEP studies (Figure 1). The main themes were related to sport and exercise as a tool for promoting education, social support and health (Figures 5, 6). It seems that the academic studies are devoted to examining physical activity and exercise as a means to promote well-being, quality of life and the related psychological aspects (Mandolesi et al., 2018). Since the psychological benefits from exercise programs are still under dispute, a growing number of professionals are seeking to study the relationship between them.

The increasing demand for improved sports performance has directed researchers’ attention to the understanding of psychological factors related to performance and success, considering that the progress in physical, technical and tactical aspects of training have fallen short of ensuring consistent successful outcomes (Stefanello, 2009). Hence, most research themes were related to football (Cluster 3, Figure 4 and Cluster 2, Figure 5) (Andrade et al., 2015). It also showed a growing interest in SEP studies in some specific sports modalities such as football, indoor football, volleyball and tennis.

Nevertheless, volleyball has been identified as the most investigated sport in sports science scientific journals published in Portuguese, while football was the second most studied sport (Dominski et al., 2018). It is interesting in a country known for its football performance. On the other hand, the performance of the Brazilian volleyball teams may have driven such interest. The motivation was the most common word, as also evidenced by Vieira et al. (2013) while analyzing the SEP themes presented in Brazilian Congresses between the years 2010 and 2012. However, research involving motivation seems to be calling less attention to current sport and exercise research (Table 1 and Figure 4). On the other hand, stress, anxiety, coping strategies, burnout and overtraining have gained focus as a significant number of researchers that are trying to understand how athletes face pressure and adversities in the context of sport’s performance. It seems that performance-related aspects of emotional control are a promising topic (Stefanello, 2009). The use of psychophysiological markers (e.g., cortisol) may reveal the researchers’ efforts toward more objective measurements and also indicate the need to integrating different areas of knowledge to promote deeper advances in science. The role of coaches for athletes’ psychological aspects received far less attention.

Research involving transcultural adaptation and validation of psychometric instruments showed a growing trend (Figure 4, Cluster 4), as they are of scientific relevance. Indeed, these instruments were first published in English, which is a limiting factor for researchers from other non-native English speakers. The reduced number of tools specifically designed for studying sport and physical activity may cause bias and increase eventual errors (Brandão, 2007). Thus, validating psychometric instruments (e.g., Albuquerque et al., 2017; Moreira et al., 2018; Contreira et al., 2019) is a critical issue for scientific progress in the field of SEP, especially in Brazil (Németh et al., 2016; Queiroz et al., 2016; Dominski et al., 2018).

The most studied SEP theme in Psychology was physical activity, sport and health (Figure 5). There are arguments that SEP research has been devoted to the understanding of individuals’ behavior related to exercise practice and its outcomes for health, well-being and quality of life (Gouveia, 2001; Rubio, 2018) rather than sport’s performance. Since the ‘90s, these aspects have gathered attention from psychology researchers, in an attempt to shed light on mental health, positive emotions, well-being and sense of pleasure through the practice of physical activities and exercises (Gouveia, 2001; Rubio, 2018). Hence, the present results indicate that Psychology GPs are following the trends for training methods in physical activity and its health and psychological consequences according to their particular characteristics (e.g., elderly, workers, patients, etc.).

When physical activity is considered as a tool for education (Cluster 2 and 3, Figure 5), sports practice has received attention for the development and teaching-learning process of children and adolescents in school environments. In these cases, psychology studies have focused on sports teaching as a ludic/playful activity, especially in team sports. Psychology programs were also concerned with constructing and validating psychometric scales to assess a variety of aspects that influence athletes’ emotions and performance (Figure 5, Cluster 2). A study by Féres-Carneiro et al. (2010), found that research on methods and assessment tools were most evident in the areas of organizational and work psychology, with apparent gaps for legal and environmental psychology. In sports, the first studies adopted instruments from studies involving clinical psychology scenarios. The advances in the research provided by the GPs have helped a better methodological approach while studying sports performance (Rubio, 2018).

The contribution of the psychologists’ in the context of the sport is related to social development (Cluster 2, Figures 5, 6). Sports practice can be seen as a way to promote socialization and social engagement (Rubio, 1999). Furthermore, the use of sports in social contexts may encourage psychologists to work toward identifying formation and social transformation (Vissoci et al., 2018).

Despite the findings found in the present study, some limitations deserve attention. The first aspect of being considered is that it was impossible to include all researches involving SEP that were not registered in governmental databases (i.e., Sucupira Platform). The fact that some thesis and dissertations may have developed some aspects further than those identified in the present study (as secondary publications) cannot be ruled out. Finally, the present study is limited to the data available up to 2016, and more recent studies were not included. Large databases including updated periods are difficult to obtain. Future research is required to identify if these trends are still present in more recent years.

## Conclusion

Analyzing the themes used in the field of SEP in Exercise and Sport Science and Psychology graduate programs revealed that the majority of SEP researches are performed in Exercise and Sport Science programs. These studies have focused on the effects of physical exercise on life quality. Psychology graduate programs have shown significant contributions to SEP by approaching the sport as a tool for promoting health, education and social support.

### Practical Implications

The present findings may contribute to the understanding and the discussion of the themes being investigated in the field of SEP, showing the lack of researches guiding in this field. As practical implications, it is highlighted the need for contemporary themes should be studied in sports, in which sports practice appears to be a promising area that received much less attention than physical activity and exercise.

## Data Availability Statement

This data can be found here: https://doi.org/10.6084/m9.figshare.9883595.v1.

## Author Contributions

LF, AR, and JS conceived the original idea of the study. NC, CM, and JV selected the researches and contributed to data processing and analysis. JV analyzed and presented the data. LF, JS, NC, CM, AR, LSF, and AL wrote and organized the manuscript. All authors reviewed the document and approved the final version for submission.

## Conflict of Interest

The authors declare that the research was conducted in the absence of any commercial or financial relationships that could be construed as a potential conflict of interest.

## Footnotes

1 https://sucupira.capes.gov.br/sucupira/public/index.xhtml
